# A study on the photocatalytic degradation performance of a [KNbO_3_]_0.9_-[BaNi_0.5_Nb_0.5_O_3−*δ*_]_0.1_ perovskite

**DOI:** 10.1039/c9ra07310h

**Published:** 2020-01-08

**Authors:** Duoying Zhang, Shan Lv, Zhi Luo

**Affiliations:** Department of Electronic Engineering, Jinan University Guangzhou 510632 Guangdong Province China zhluocn@gmail.com

## Abstract

In this study, [KNbO_3_]_0.9_-[BaNi_0.5_Nb_0.5_O_3−*δ*_]_0.1_ (KBNNO) perovskite powder was synthesized *via* a conventional solid-phase reaction method. The crystal structure of the KBNNO powder was characterized on an X-ray diffractometer. The size and surface morphology of the particles were investigated *via* field emission electron scanning microscopy (FE-SEM). The KBNNO powder particles were stacked from a smooth flat layer. The photocatalytic activity of KBNNO was investigated using a methylene blue (MB) aqueous solution as a model organic substrate. The results showed that the KBNNO powder has excellent photocatalytic degradation performance. The effects of catalyst loading and the initial concentration of the MB solution on the photocatalytic activity were also investigated in this study. The experimental results proved that both catalyst loading and the initial dye concentration are important factors affecting photocatalytic degradation. As the catalyst loading increases, the photocatalytic activity increases. However, the growth rate of the degradation efficiency gradually decreases. Also, the degradation efficiency gradually decreased with the initial concentration of MB.

## Abstract 2

In this study, [KNbO_3_]_0.9_-[BaNi_0.5_Nb_0.5_O_3−*δ*_]_0.1_ (KBNNO) perovskite powder was synthesized *via* a conventional solid-phase reaction method.

## Graphical abstract

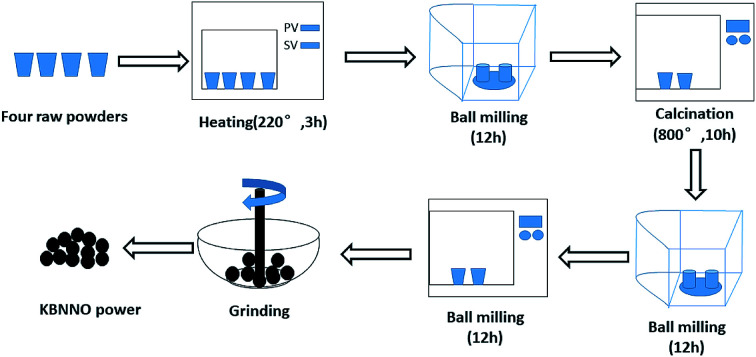

## 1. Introduction

In recent years, protecting the environment has become a high priority. The ability of perovskites to degrade dyes^1–3^ and toxic organic pollutants^4^ has attracted great research interest. The preparation process of perovskite is simple and controllable; however, most perovskites have a wide bandgap (*E*_g_ > 2.7 eV),^5,6^ and only a small amount of sunlight power can be absorbed, which seriously hinders the development of perovskites in photocatalysts. Therefore, it is necessary to develop new nontoxic photocatalysts with narrow bandgaps.

The KNbO_3_ (KNO) perovskite is a stable photocatalyst with an ABO_3_ structure.^7–9^ In general, larger rare-earth metal cations are located in the A-site, while smaller transition metal cations are located in the B-site. The excitation across the bandgap is essentially a charge transfer from the oxygen (O) 2p states at the valence band maximum to the transition-metal d states at the conduction band minimum.^5^ The hybridization between the titanium 3d states and the oxygen 2p states is essential for the ferroelectricity in BaTiO_3_ and PbTiO_3_, as proved by Cohen *et al.*^10^ The element at position B was shown to play an important role in the ferroelectric properties of perovskite,^5,7,10,11^ and can change the optical band gap and introduce a tail state. KNO has a wide bandgap of 3.2 eV, which limited its performance. Chemical element doping is one of the effective ways to change the physical and chemical properties of a material.

Grinberg *et al.* successfully incorporated nickel into the B-site of KNO, and the optical properties of KBNNO were improved while still retaining ferroelectricity. The direct band-gap of KBNNO can be as low as 1.39 eV and is much smaller than the parent KNO perovskites. Thus, most sunlight energy can be utilized by KBNNO.^5–7^ This breakthrough aroused great interest about the KBNNO perovskite. Doping Ni element into KNbO_3_ crystal, with one cation driving ferroelectricity and another giving an *E*_g_ in the visible range. It was mixed with BaNi_0.5_Nb_0.5_O_3−*δ*_ (BNNO) to introduce a combination of Ni^2+^ on the B-site and oxygen vacancy. The K^+^ cations in the A-site were replaced by the Ba^2+^ cations to compensate the charge, and the large sizes of the K^+^ and Ba^2+^ cations favor solubility and vacancy formation. This will help the perovskite remain stable. However, little research has been done on the photocatalytic performance of KBNNO.

In this article, KBNNO powder was successfully prepared *via* a conventional solid-phase reaction method, and the photocatalytic degradation performance was investigated. The results showed that the KBNNO perovskite had excellent photocatalytic degradation performance, and the net photocatalytic degradation efficiency could reach about 40% in 2 hours. The catalyst loading and degraded substance concentration are important factors affecting the photocatalytic degradation efficiency of the KBNNO powder.

## 2. Experiment

### 2.1 Preparation

**Fig. 1:**
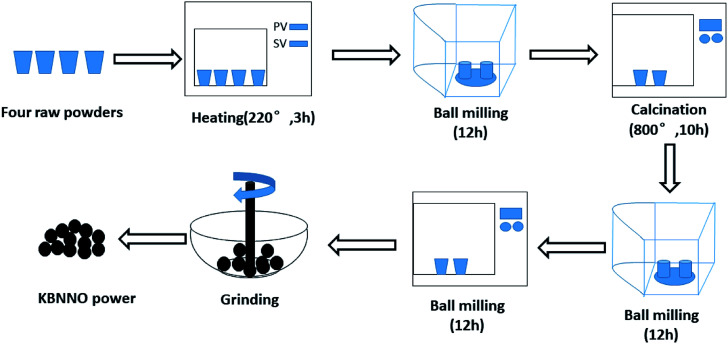
The preparation process of KBNNO.

The KBNNO powder was synthesized *via* the traditional solid-phase reaction method. The preparation process of the KBNNO powder is shown in Fig. 1. Potassium carbonate (K_2_CO_3_), cesium carbonate (BaCO_3_), nickel oxide (NiO), and antimony pentoxide (Nb_2_O_5_) were used as raw materials (all drugs were of analytical grade). The KBNNO powder was prepared by ball milling twice and undergoing the high-temperature calcination process twice. It was ground into a fine powder with an agate mortar for the next experiment.

### 2.2 Characterization

The crystal structure was characterized using an X-ray diffractometer (Bruker D8-Advance). The surface topography and crystal size of the sample were analyzed using a field emission scanning electron microscope (ZEISS, ULTRA-55 type). The Raman spectrum (excitation wavelength is *λ* = 532 nm) was used to characterize the doping and ferroelectricity performance. The absorbance of the MB solution was measured using an ultraviolet-visible spectrophotometer (UV-2600) to investigate the photocatalytic degradation performance of the KBNNO powder.

### 2.3 Photocatalytic degradation experiment

The photocatalytic efficiency of the KBNNO powder was investigated by the degradation of MB at room temperature. During each experiment, the experiment was performed under the illumination of a high-pressure mercury lamp (GGY250W, main peak wavelength 365 nm, energy 3400 MJ cm^−2^, power 250 W), which was about 25 cm above the surface of the dye. A 100 mL volume of MB solution (20 mg L^−1^) was poured into a 250 mL beaker without the lid, and then 150 mg of KBNNO catalyst was added. The suspension was ultrasonically oscillated for 10 min, and then magnetically stirred (800 rpm) for 30 min to achieve adsorption equilibrium.^12^ Both processes were done in a dark environment. After that, the light source was turned on. A 4 mL volume of the upper suspension was pipetted every 30 min and immediately centrifuged (10 000 rpm, 5 min), A 3 mL volume of the supernatant was taken to measure the absorbance of the solution. The whole photocatalytic degradation experiment lasted for 150 min.

In the experiment of investigating the relationship between catalyst loading and the KBNNO photocatalytic degradation performance, different quality catalysts (120 mg, 140 mg, 160 mg, 180 mg, and 200 mg) were added to a 100 mL volume of MB solution (20 mg L^−1^). In the study of the influence of the MB solution concentration on the photocatalytic performance of the KBNNO powder, 150 mg of KBNNO powder was added to different concentrations of MB solution (10 mg L^−1^, 15 mg L^−1^, 20 mg L^−1^, 25 mg L^−1^, and 30 mg L^−1^). The natural degradation of MB under illumination was measured in the same way.

**Fig. 2:**
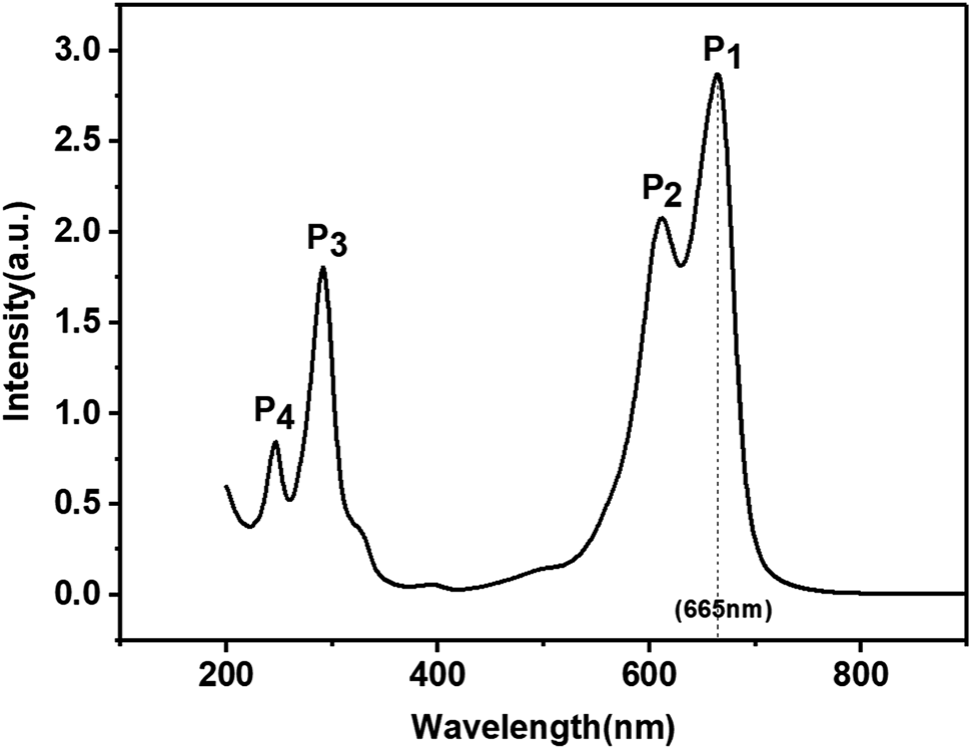
The UV-visible absorption spectrum of the MB solution.

The UV-visible spectrum of the MB solution is shown in Fig. 2. There are four absorption peaks at 246 nm, 292 nm, 612 nm, and 665 nm, respectively. The maximum absorbance intensity of the MB solution occurred near 665 nm. Therefore, the absorbance intensity of this wavelength was selected to calculate the photocatalytic degradation rate (De). The absorbance of the MB solution before and after photoreaction was measured by UV-visible spectrophotometry, and then the degradation rate (De) of MB was calculated according to the following formula:^1,3^1
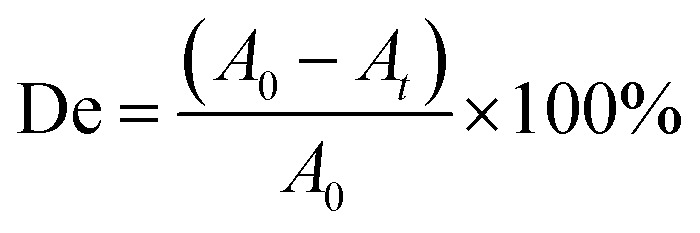


In formula (1), *A*_0_ represents the initial absorbance of the MB solution, which was measured after adsorption equilibrium and before the light was turned on, and *A*_*t*_ represents the absorbance of the MB solution after *t* minute.

## 3. Results and discussion

### 3.1 Structural characteristics of KBNNO powders

**Fig. 3:**
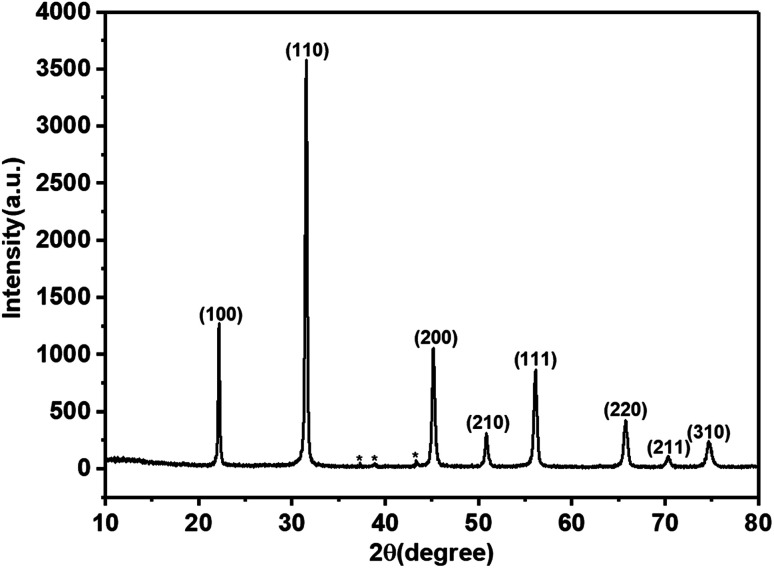
XRD pattern of KBNNO powder (NiO peaks are marked by an asterisk).

The XRD pattern of the KBNNO perovskite powder is shown in Fig. 3. The main diffraction peaks of the sample appeared at 22.156°, 31.515°, 45.125°, 50.8263°, 56.077°, 65.702°, 70.360°, and 74.630°, while very weak NiO peaks were also observed. It is consistent with Grinberg's work.^5^ All of the peaks of KBNNO were very narrow and sharp, indicating the presence of big particle sizes and high crystallinity.^13^ According to the XRD, the sample had a tetragonal structure (*a* = 4.016 Å, *b* = 4.016 Å, *c* = 3.587 Å) with a *P*4_2_*cm* space group, while KNO had an orthorhombic structure (*a* = 5.695 Å, *b* = 5.7213 Å, *c* = 3.9739 Å). There was a decrease in the lattice parameters of *a* and *c*. This variation is consistent with the transition from an orthorhombic to a weak tetragonal structure, and is similar to the work of Zhou *et al.*^7^ The decreases in the *b* lattice parameter may be due to the presence of NiO impurities. The change of the phase is similar to results reported by Grinberg *et al.*^5^ and Zhou *et al.*^7^ In their works, they reported that the increase of the composition *x* in KBNNO between *x* = 0 and *x* = 0.4 shows a gradual transition from an orthorhombic to cubic structure, which revealed a weakly tetragonal structure at *x* = 0.1.^7^

**Fig. 4:**
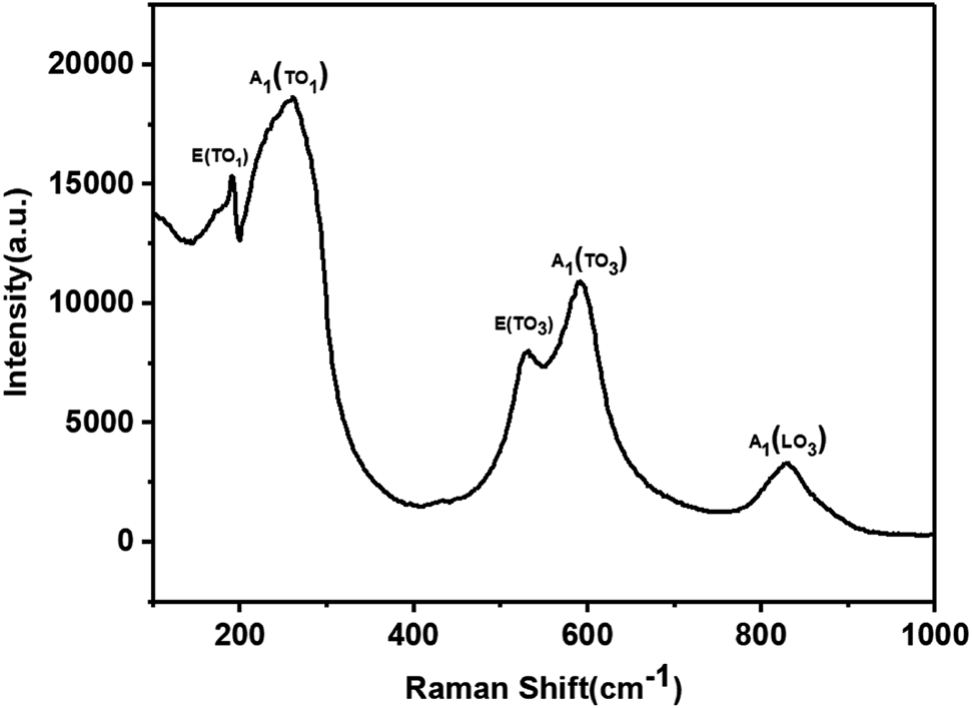
Raman spectrum of KBNNO powder.

Raman scattering is very sensitive to changes in lattice vibration. It was used to investigate the effects of Ni^2+^ and Ba^2+^ doping (Fig. 4). There are two distinct Raman peaks in the low wavenumber region (100 cm^−1^–400 cm^−1^) and three characteristic Raman peaks in the high wavenumber region (500 cm^−1^–900 cm^−1^). These results were consistent with previous research results.^4,6,7^ In the high wavenumber region, vibrations associated with the oxygen octahedra are present. The two peaks at 188 cm^−1^ and 245 cm^−1^ are indicative of a long-range polar order, as seen by Luisman *et al.*^14^ The resonance depth at 200 cm^−1^ and the peak at 820 cm^−1^ can also be observed. The previous study suggested that these peaks come from the ferroelectricity in the KNbO_3_-based solid solutions.^6,15^ The results showed that the KBNNO is ferroelectric at room temperature.

**Fig. 5:**
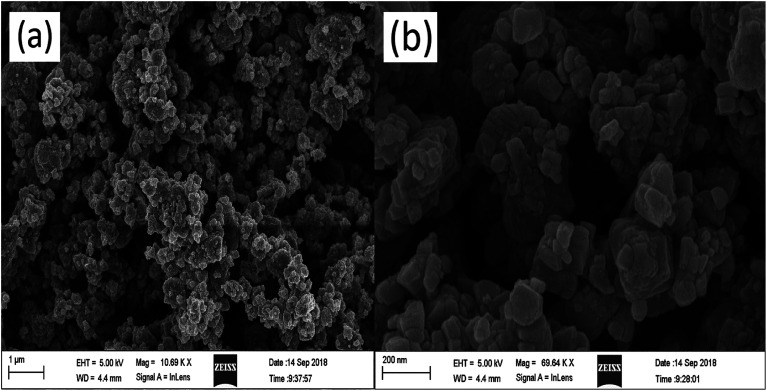
FE-SEM image of KBNNO powder.

The sample morphology was characterized by FE-SEM (Fig. 5). KBNNO powder is composed of many different sizes of particles, and there are large gaps between the particles. All particles were observed to be nanocrystalline powders, but the particle size varied widely. The surface of the particles is smooth and flat. The smaller particle size of the catalyst, the larger surface area, and the presence of more active sites are all more favorable properties for the photocatalytic degradation reaction.

### 3.2 Photocatalytic activity measurement

**Fig. 6:**
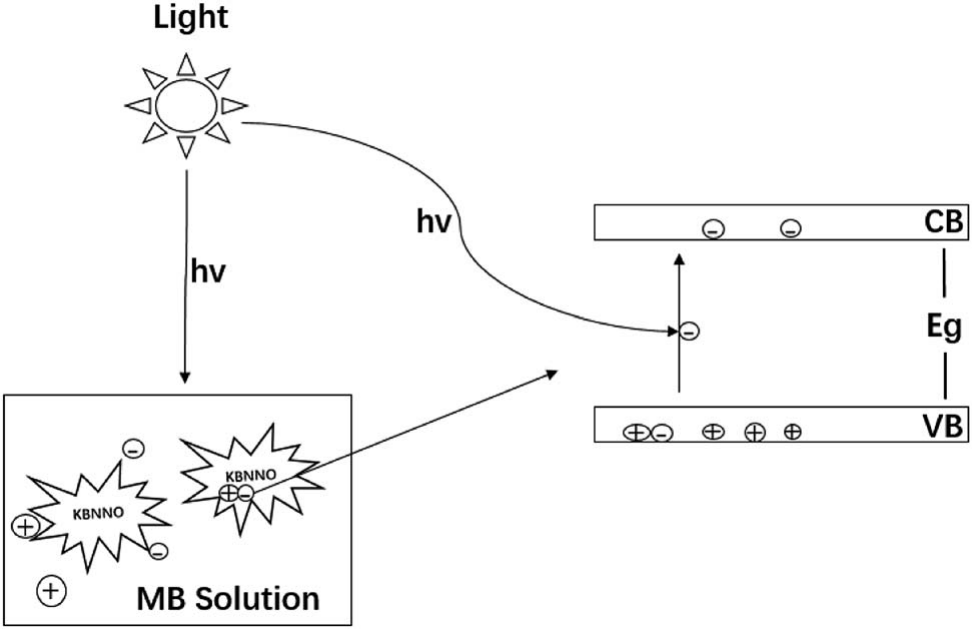
Schematic diagram of the KBNNO photocatalytic degradation of the MB solution.

The schematic diagram of the photocatalytic degradation of the MB solution by the KBNNO perovskite is shown in Fig. 6. When the catalyst is illuminated with light consisting of an energy that is equal to or greater than the bandgap, the electrons are excited from the valence band to the conductor band, and electron–hole pairs are generated. Separated electrons and holes diffuse to the surface of the catalyst and react with water, a hydroxyl group and molecular oxygen adsorbed on the surface of the catalyst, producing reactive radicals that can induce the mineralization of organic pollutants.^16,17^

**Fig. 7:**
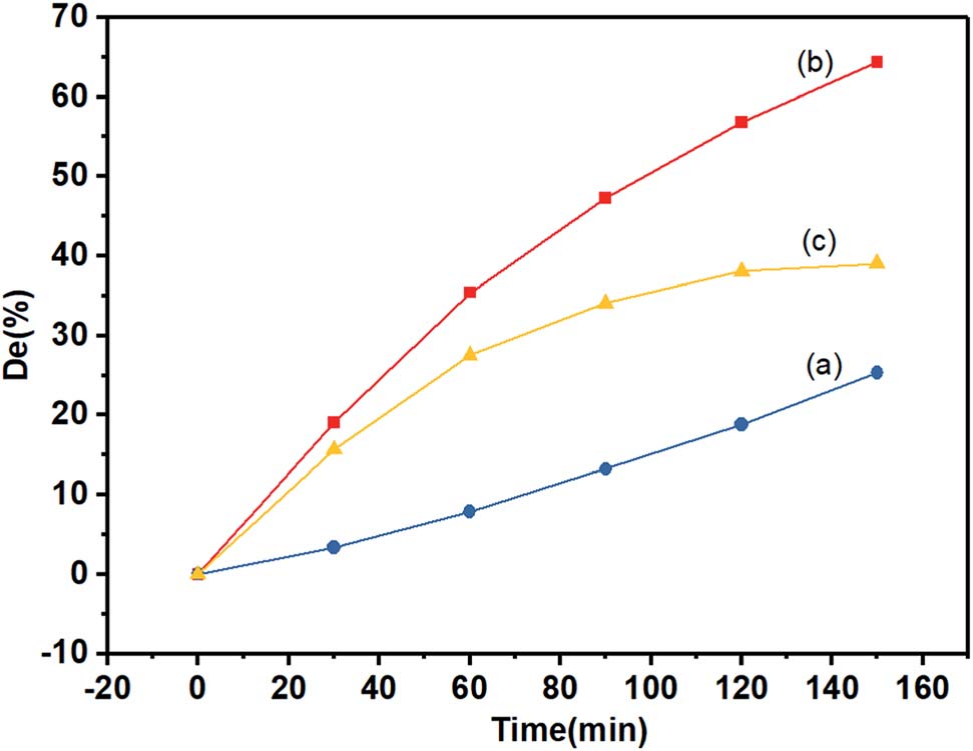
The degradation of the MB solution with irradiation: (a) without KBNNO; (b) with KBNNO; (c) net catalytic degradation efficiency.

The photocatalytic degradation efficiency of KBNNO is shown in Fig. 7. Without the KBNNO powder, the MB solution was almost linearly decomposed, and the degradation efficiency was only about 15% (Fig. 7(a)). With the KBNNO powder, the degradation efficiency increased rapidly, and the degradation efficiency reached 55% in 120 min (Fig. 7(b)). Curve (c) is the net photocatalytic degradation efficiency curve of KBNNO, which is obtained by subtracting curve (b) from curve (a). The degradation efficiency first increases, and then stabilizes gradually. The stabilization of the degradation efficiency is caused by the compounds formed during the dye degradation process. During the process, the competition between the intermediate and the parent molecules slowed down the degradation rate.^18^ The photocatalytic performance of KBNNO was significantly improved compared to the undoped perovskite matrix.^19^ The photocatalytic efficiency is determined by the ratio of the electron mobility to the electron–hole recombination ratio.^20^ The inhibition of photo-induced electron–hole pair recombination can improve the photocatalytic efficiency. With the doping of Ni^2+^ and Ba^2+^, a large number of oxygen vacancies exist on the KBNNO surface to keep the charge balance.^6,21–23^ During the photocatalytic reaction, oxygen vacancies can capture photoelectrons as electron traps and prevent electron–hole recombination. In addition, a large number of oxygen vacancies will cause a strong absorption of OH groups on the catalyst surface. The high concentration of OH groups adsorbed on the surface of the KBNNO grains will capture more photogenerated holes, and can effectively inhibit the recombination of photoinduced electron–hole pairs.^24^

### 3.3 Influence of catalyst loading

**Fig. 8:**
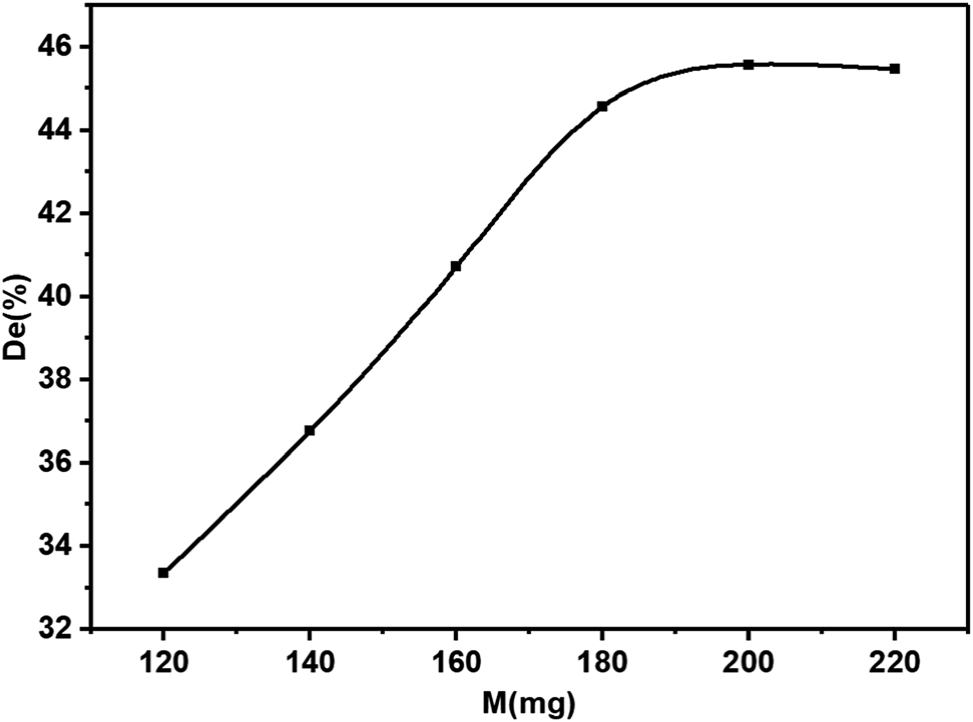
The relationship between the KBNNO catalyst loading and degradation efficiency.

Catalyst loading is one of the main parameters affecting the degradation efficiency. In order to avoid the abuse of catalysts, it is necessary to find the optimum loading for effective dye removal. The effect of catalyst loading on the photodegradation of dyes was investigated (Fig. 8). The results showed that with the increase of catalyst loading, the photocatalytic efficiency first increased linearly and stabilized gradually. When the catalyst-loaded KBNNO powder was 120 mg, the degradation efficiency was 33%. In addition, the degradation efficiency reached 44%, while the loading increased to 180 mg. However, when the loading was 200 mg, the degradation efficiency was only 45%. This result is similar to that found in other photocatalyst studies.^18,25,26^ This result can be explained as follows: when the catalyst load is small, the catalyst absorbs fewer photons for the photocatalytic reaction, and thus the photocatalytic activity is lower. As the catalyst loading increases, the number of photon absorption centers and activity centers on the catalyst surface increases, thereby increasing the activity of the catalyst. However, as the catalyst loading increases, the number of photons tends to be saturated. Increased catalyst loading may cause a light blockage. This will affect the photocatalytic efficiency, and will also result in the wasted catalyst.^1^ The loading of the KBNNO catalyst is 180 mg, which is the most suitable catalyst loading under the experimental conditions. The catalytic effect is obviously improved, and the required catalyst loading is less.

### 3.4 Relationship between the initial dye concentration and degradation efficiency

**Fig. 9:**
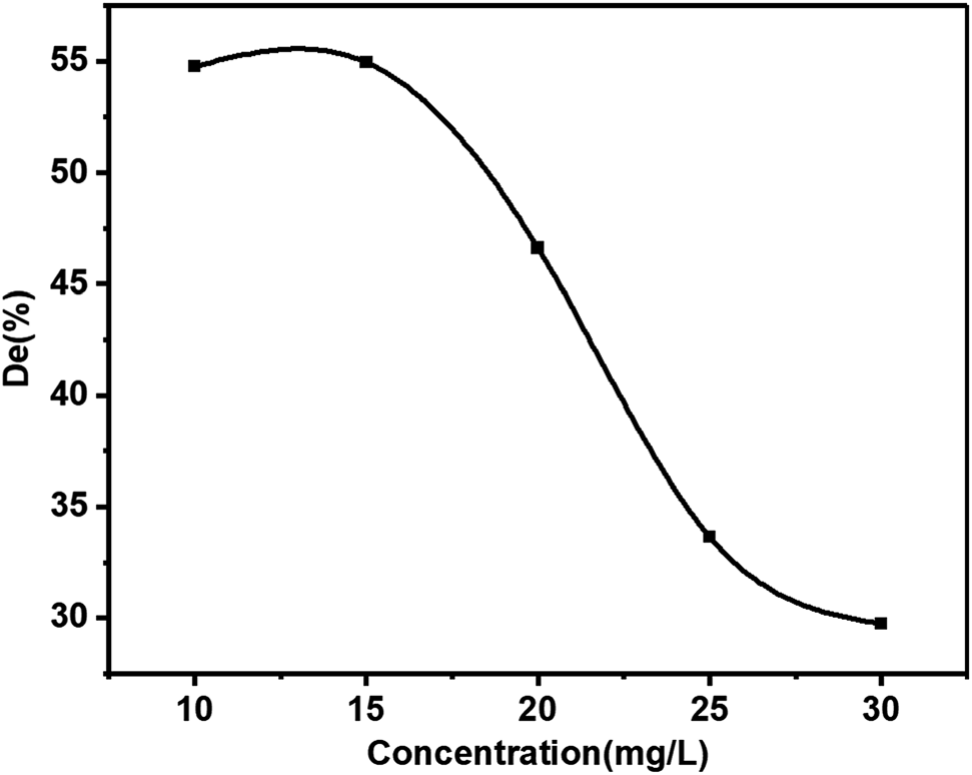
The effect of the initial concentration of the MB solution on the catalytic efficiency.

Contaminant concentration is a very crucial parameter in wastewater treatment.^18^ The relationship between the initial dye concentration (10 mg L^−1^, 15 mg L^−1^, 20 mg L^−1^, 25 mg L^−1^, and 30 mg L^−1^) and the photocatalytic degradation efficiency was studied (Fig. 9). The photocatalytic degradation efficiency of KBNNO hardly changed with the increase of the MB solution concentration while the initial concentration was less than 15 mg L^−1^. This indicated that the KBNNO maintained a stable catalytic performance in a low concentration of the degraded substance. With the increase of the initial concentration of the MB solution, the photocatalytic efficiency of KBNNO was gradually reduced, and the photocatalytic degradation of other dyes have shown similar results.^1,12,27–29^ When the initial concentration of MB was 10 mg L^−1^, the degradation efficiency of the KBNNO powder was 55%. The degradation efficiency was only 30% when the concentration of the dye was 30 mg L^−1^. These results can be explained as follows: (1) as the initial concentration of the dye increases, the degradation efficiency decreases significantly. This is because the substrate has a strong absorption of light in the wavelength range in which the catalyst is excited, which may reduce the catalytic efficiency;^12,30^ (2) when the initial concentration of the dye increases, the amount of dye adsorbed on the catalytic surface increases, which hindered the catalyst from absorbing photons to generate electron–hole pairs, thereby reducing the degradation efficiency; (3) the increase of the dye concentration also increased the path length of the photons entering the dye solution.^29^ The photocatalytic degradation efficiency of KBNNO is stable at low concentrations, and decreases rapidly with increasing dye concentration.

## 4. Conclusions

In summary, a KBNNO perovskite powder with good crystallization properties was prepared *via* a conventional solid-phase reaction method. The perovskite KBNNO had excellent photocatalytic degradation properties, which were shown by the degradation experiments. It was proved that the catalyst loading and the initial dye concentration are very effective factors for affecting the photocatalytic efficiency of perovskite. Other factors that affect the photocatalytic efficiency of KBNNO perovskites require further research.

## Conflicts of interest

There are no conflicts to declare.

## Supplementary Material
